# Reconstruction of the On-Top Two-Electron Density from Natural Orbitals and Their Occupation Numbers

**DOI:** 10.1021/acs.jctc.5c00024

**Published:** 2025-04-08

**Authors:** Jerzy Cioslowski, Krzysztof Strasburger

**Affiliations:** † Institute of Physics, University of Szczecin, Wielkopolska 15, 70-451 Szczecin, Poland; ‡ Max-Planck-Institut für Physik komplexer Systeme, Nöthnitzer Straße 38, 01187 Dresden, Germany; § Department of Physical and Quantum Chemistry, Faculty of Chemistry, Wrocław University of Science and Technology, Wybrzeże Wyspiańskiego 27, 50-370 Wrocław, Poland

## Abstract

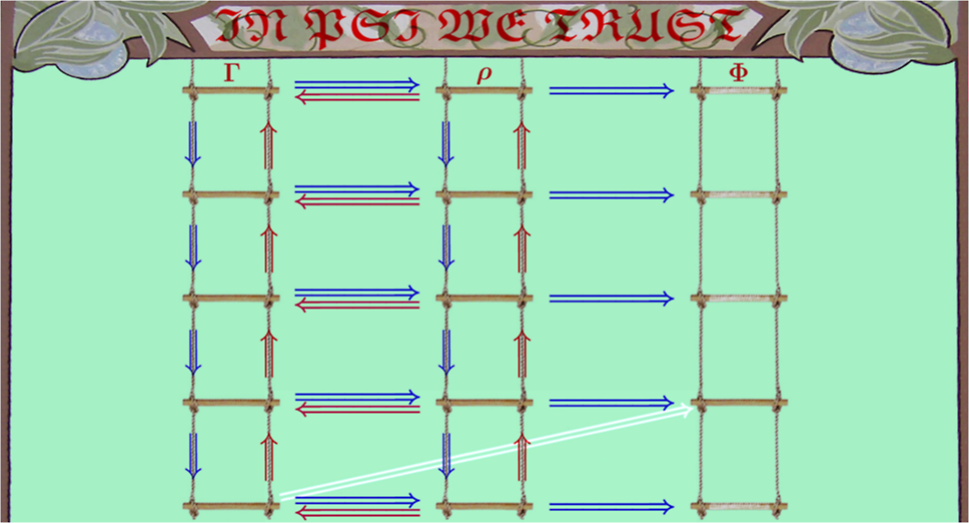

Spatial derivatives of the natural orbitals (NOs) at their nodal surfaces are shown to encode information about the on-top two-electron density Φ_2_(*r⃗*) in an approximate manner. This encoding, which becomes exact at the limit of an infinite number of nodal surfaces, allows the reconstruction of Φ_2_(*r⃗*) up to a multiplicative constant that can be retrieved from an identity involving the NO in question and its occupation number. This reconstruction provides a new consistency check for electronic structure formalisms, such as the one-electron reduced density matrix theory, that employ NOs as primary quantities.

## 1 Introduction

Within the Born–Oppenheimer approximation, an *N*-electron atom or molecule is just an assembly of Coulombically interacting fermions whose nonrelativistic description is provided by a single-component wave function Ψ(***x***_1_, ..., ***x***_*N*_) [here and in the following, the atomic units and the standard notation  for the combined spatial and spin coordinates are used; the normalization ∫...∫ |Ψ|^2^ d***x***_1_ ... d***x***_*N*_ = 1, where ∫d***x***_*k*_ stands for , is assumed]. However, due to its complexity rising rapidly with *N*, Ψ(***x***_1_, ..., ***x***_*N*_) seldom plays the central role in the formalisms of quantum chemistry. Instead, these formalisms usually involve derivative quantities, such as reduced density matrices, densities, and orbitals, whose dimensionalities are *N*-independent. One of these quantities is the on-top two-electron (a.k.a. pair) density , defined as
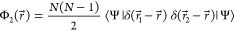
1where  is the Dirac delta. Since its initial employment in the resolution of the symmetry dilemma in the description of spin-polarized systems within the density functional theory (DFT),^1^ Φ_2_ has been steadily gaining prominence in various facets of the electronic structure theory.

Measuring the probability density of finding two electrons at their coalescence, Φ_2_ and its normalization
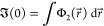
2i.e., the intracule density  at  = 0, are exceptionally sensitive to the electron correlation effects. This sensitivity is vividly illustrated by the calculations for the ground states of H^–^, He, Li^+^, Be^2+^, Li, and Be, which at the HF/6-311G level of theory produce the respective values of  equal to 0.0163, 0.1905, 0.7697, 1.9891, 0.7841, and 2.0981 that grossly deviate from their “almost exact” counterparts of 0.0027, 0.1063, 0.5337, 1.5229, 0.5426, and 1.6069.^2^ It should be emphasized that the errors in  reflect the global differences between the approximate and exact Φ_2_, the respective local errors being obviously much higher. Approximate electron-correlation theories based upon Slater determinants do not offer a facile reduction of these deviations. First of all, being incapable of reproducing the electron-electron coalescence cusp,^3^ they fail to yield accurate Φ_2_ unless very large basis sets are used. This failure is observed even for the simplest systems such as the He atom, for which ground-state values of  calculated at the MP2/20s, MP2/20s10p, and MP2/20s10p10d levels of theory read 0.1606, 0.1300, and 0.1184, respectively.^4^ Second, due to the short-range nature of the electron-electron coalescence cusp, approaches geared towards the description of nondynamical correlation effects (such as MCSCF with a small active space) produce only minor improvements in the accuracy of the computed Φ_2_. For this reason, the quantities recently employed in the multiconfigurational generalizations of DFT^5^ and in the context of machine learning^6^ are only crude approximations to Φ_2_. Consequently, they should be regarded as model rather than genuine on-top two-electron densities.

These considerations underscore the importance of researching the viability of possible alternatives to direct evaluation of the on-top two-electron density from its definition 1 (either as the expectation value or the corresponding energy derivative^4^). One such alternative, built around a recently derived off-diagonal cusp condition,^7^ is investigated in this paper that, in order to demonstrate the uniqueness of the proposed approach, commences with a brief review of the interrelations among various reduced density matrices and densities. Next, the difficulties that arise upon the application of the cusp condition in question to wave functions constructed from Gaussian basis functions are first explained and then resolved. The remarkable simplification of the resulting prescription for the reconstruction of Φ_2_ in the presence of spherical symmetry is described. These theoretical developments are followed by several numerical tests. Finally, the strengths and limitations of the novel approach to the computation of Φ_2_ are briefly discussed.

## 2 Theory

A brief elucidation of the interrelations among various quantities derived from the electronic wave function facilitates the understanding of the proposed formalism and highlights its unique features. These interrelations are faithfully depicted by a set of three parallel ladders, each comprising *N* rungs. Moving from the top to the bottom, the first ladder is populated with the density matrix ^*N*^Γ ≡ ^*N*^Γ(***x***_1_, ..., ***x***_*N*_; ***x***_1′_, ..., ***x***_*N*′_) = Ψ*(***x***_1′_, ..., ***x***_*N*′_) Ψ(***x***_1_, ..., ***x***_*N*_) followed by its reduced counterparts^8^
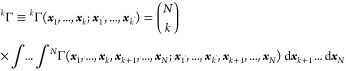
3with *k* = *N* – 1, ..., 1. Within the second ladder that comprises the densities, ρ_*N*_ ≡ ρ_*N*_(***x***_1_, ..., ***x***_*N*_) = |Ψ(***x***_1_, ..., ***x***_*N*_)|^2^ occupies the top rung, while those involving fewer than *N* particles, defined as
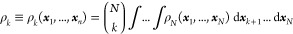
4are assigned successively to the rungs below it according to descending *k*. The third ladder is that of the (spinless) on-top densities, simply given by
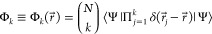
5As Φ_1_ is equal to the spin-summed ρ_1_ [i.e., Φ_1_(*r⃗*) = ∫ρ_1_(***x***_1_)δ(*r⃗*_1_ – *r⃗*) d***x***_1_] and  for all *k* ≥ 2, this ladder has only one rung of relevance, namely that corresponding to Φ_2_.

The above definitions imply both the intraladder interrelations, namely
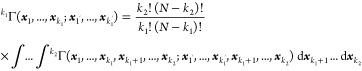
6and
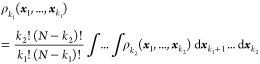
7and the interladder ones, namely
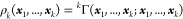
8and
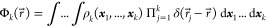
9

**Figure 1:**
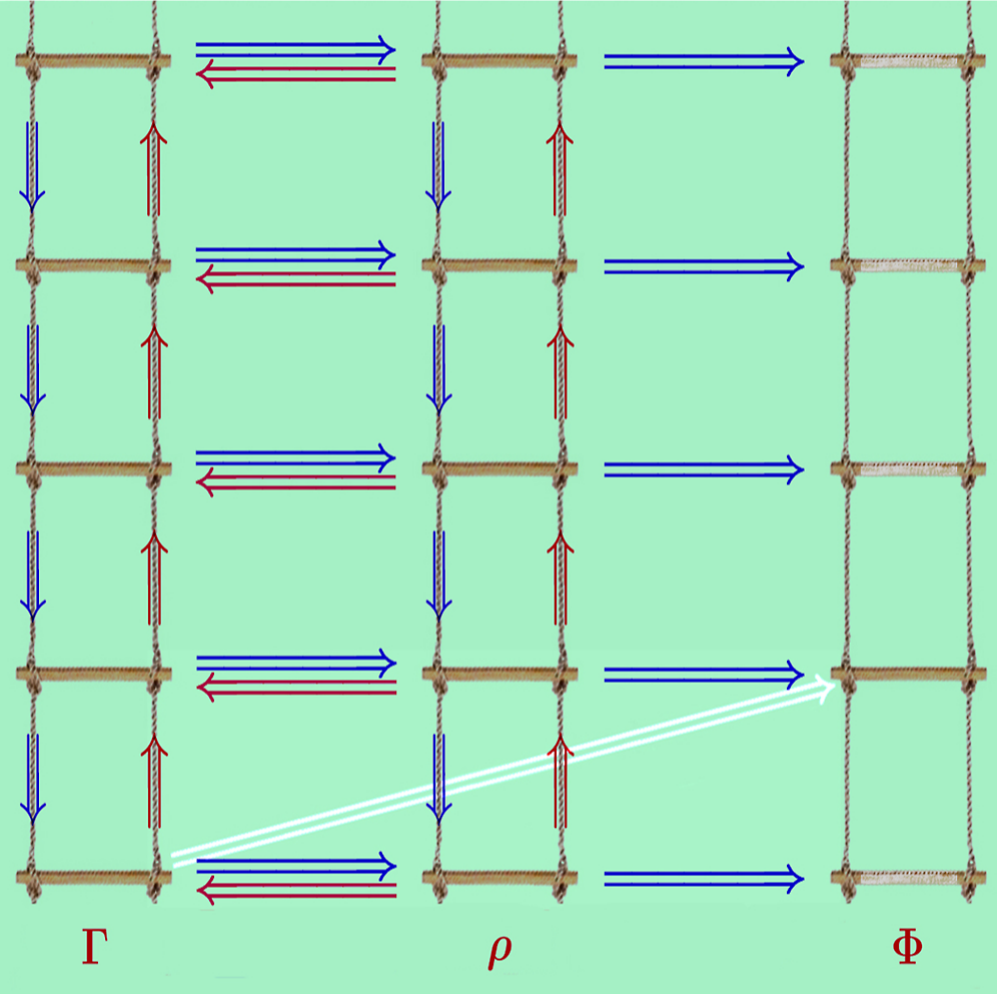
The three ladders depicting the interrelations among the reduced density matrices, the densities, and the on-top densities.

The relationships defined by the contractions 6 and 7 are both explicit and unidirectional, i.e., ^*k*_2_^Γ ⇒ ^*k*_1_^Γ and ρ_*k*_2__ ⇒ ρ_*k*_1__ for 0 < *k*_1_ < *k*_2_ < *N* only. The same is true for the relationships 8 and 9 that link the *k*-matrix, the *k*-particle density, and the on-top *k*-particle density through the chain ^*k*^Γ ⇒ ρ_*k*_ ⇒ Φ_*k*_ for all 1 ≤ *k* ≤ *N*. In all of these relationships, the unidirectionality requirement cannot be abrogated as, e.g., there exist infinitely many proper (i.e., properly normalized and permutationally antisymmetric) wave functions producing the *N*-particle densities that, upon being contracted according to eq 4, yield a given *N*-representable (i.e., nonnegative-valued and normalized to *N*) one-particle density .^9^ However, any *N*-representable ρ_1_ can be assigned a unique (up to a phase factor and a possible degeneracy) Ψ by selecting from among these wave functions the one minimizing the expectation value of the operator  (where  involves differentiation with respect to the components of ). Invoking the actual form of the interparticle interactions, this prescription for linking Ψ with ρ_1_, which is the essence of the celebrated Levy-Lieb constrained search,^10,11^ has the important property of furnishing the wave function of a nondegenerate ground state from its one-particle density. It allows the formulation of chains of interrelations uniquely linking any pair of quantities within the first two ladders, e.g., ^2^Γ ⇒ ρ_2_ ⇒ ρ_1_ ⇝ Ψ ⇒ ^5^Γ ⇒ ρ_5_, etc., where ⇝ denotes the assignment stemming from the constrained search. In other words, traversing the first two ladders downward or moving horizontally from a given rung of the first ladder to that of the second one is associated with closed-form interconversion identities (marked blue in Figure 1), whereas movements in the opposite directions involve implicit interconversions (marked red in Figure 1) hinging upon the ρ_1_ ⇝ Ψ assignment that cannot be expressed as an explicit formula.

In light of the above discussion, it may appear that the only other movements within the ladders producing interrelations among the quantities under study are those from the individual rungs of the second ladder to their counterparts within the third one. However, there also exists the ^1^Γ ⇒ Φ_2_ link (marked white in Figure 1). Unlike the aforementioned relationships, this link relies on the particular form of the interparticle interactions but not on the constrained search. It originates from the cusp conditions that arise from the singularities in the Coulomb potential occurring at the coalescences of the positions of two particles and at the positions of individual particles at which the confining potential (e.g., that due to nuclei) becomes infinite. The cusp conditions for Ψ^3^ propagate through the rungs of all the three ladders.^12−15^ Among the resulting identities, those for the logarithmic derivatives of the *k*-matrices, the *k*-particle densities, and the *k*-particle on-top densities with respect to their individual spatial arguments do not furnish any new interrelations between these quantities. On the other hand, the recently derived off-diagonal condition^7^
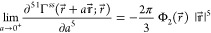
10where *s* = α or *s* = β, links the spin components  and  of the 1-matrix
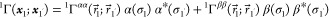
11with the on-top two-particle density . It quantifies the unsmooth aspect of the asymptotic behavior of  at the limit of  that turns out to be driven by the fifth power of the length of .

The condition 10, which is the only currently known case of a closed-form identity yielding a *k*-particle quantity from that pertaining to *k* – 1 particles, is of substantial importance to the electronic structure theory. In particular, it provides a consistency check for the 2-matrices (or, equivalently, the 2-cumulants) underlying the approximate functionals^16,17^ of the one-electron reduced density matrix functional theory (1-RDMFT).^10,18−20^

### 2.1 Reconstruction of Φ_2_ from the Natural Orbitals and Their Occupation Numbers

At the first glance, the computation of Φ_2_ from ^1^Γ via eq 10 appears to be a trivial task amounting to the evaluation of the partial fifth derivative of the latter. However, the vast majority of quantum-chemical calculations is presently based upon approximate wave functions constructed from either one-electron functions (orbitals) or explicitly correlated Gaussians.^21^ Lacking the electron-electron coalescence cusp, these wave functions produce 1-matrices that, when inserted into eq 10, yield vanishing Φ_2_. Consequently, an alternative approach to the extraction of the on-top two-electron density from the 1-matrix has to be considered.

The spin components of the 1-matrix are known to have the spectral decomposition^22^
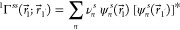
12where *s* = α or *s* = β, the functions  (which form an orthonormal set) are the natural orbitals (NOs), and {ν_*n*_^*s*^} are the respective occupation numbers (ordered nonascendingly). It follows directly from eq 12 that the NOs and their occupation numbers are, respectively, the eigenfunctions and eigenvalues of a homogeneous Fredholm equation of the second kind that reads
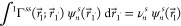
13Let  be the solution of the equation
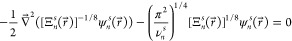
14and let . Combining eqs 13 and 14 produces
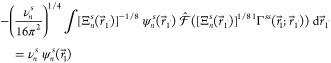
15upon integration by parts. Repeating this sequence of operations three more times yields
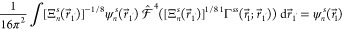
16The integral in the l.h.s. of this equation involves functions with three very different characteristics, namely the smooth but oscillatory , the (presumably smooth) nonoscillatory , and  whose fifth derivative has a discontinuity quantified by the cusp condition 10. Consequently, as the number of oscillations in  grows with *n*, the leading term in the large-*n* asymptotics of this integral is readily obtained as
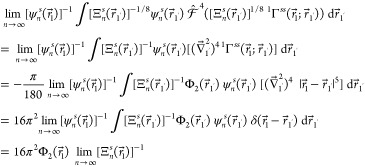
17When inserted into eq 16, this asymptotic estimate leads to the conclusion that , as expected from the previously derived zero-energy Schrödinger equation satisfied by ψ_*n*_^*s*^ and ν_*n*_^*s*^ at the limit of *n* → ∞.^23^

The above conclusion gives rise to a prescription for extracting the on-top two-electron density from the NOs and their occupation numbers that simply involves finding the solutions {} of eq 14 for various *n* and then extrapolating them to the limit of *n* → ∞. However, the nonlinearity of this equation precludes its solution by analytical means. Moreover, eq 14 is stiff, which hinders its numerical solution. Fortunately, it turns out that these difficulties can be completely circumvented by noting that at the nodal surfaces of , at which , eq 14 simplifies to
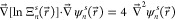
18Thus, at all the points located on all the nodal surfaces of  the logarithmic derivatives of  in the directions of the respective surface normals are readily obtainable from the gradients and the Laplacians of . As the number of these nodal surfaces grows with *n*, the distances between them diminish. Therefore, carefully devised interpolants  ≡  furnishing the approximate values of  for  at which the NOs do not vanish also yield  at the limit of *n* → ∞, allowing its reconstruction without actually solving eq 14.

### 2.2 Case of Spherically Symmetric Φ_2_

The application of the aforedescribed approach to systems with spherically symmetric  permits its detailed elucidation and facilitates the assessment of its viability. For such systems, , , , and ν_*n*_^*s*^ are replaced by Φ_2_(*r*), ,  [where  is the spherical harmonic], and . In turn, eq 18 becomes
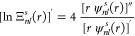
19as the nodal surfaces of  are replaced by the nodes of . Thus, for a given combination *s*, , and , the reconstruction of Φ_2_(*r*) from  proceeds through five steps. First, all the  nodes  of  are initially located and then ordered ascendingly. Second, the values of  ≡  are computed from eq 19 [with the exception of the first node at *r* = 0, for which the value of −4 *Z*, where *Z* is the charge of the nucleus, is assigned in accordance with the cusp condition for (15)]. Third, the logarithmic derivative  of the interpolant  is obtained as an explicit function of *r*. Fourth, this function is integrated, yielding  up to an integration constant. Fifth, this constant is determined, furnishing in turn the desired approximation .

The interpolation scheme employed in the third step of this procedure has to be free of Runge’s phenomenon and produce a sufficiently smooth interpolant amenable to algebraic integration. The barycentric formalism of Berrut^24^ fulfills these requirements, yielding
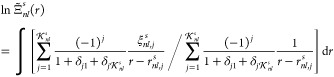
20where δ_*ij*_ is the Kronecker delta. The missing integration constant is determined from the condition
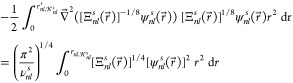
21that arises from eq 14 upon multiplication by  , integration over a ball with the radius equal to , and finally the replacement of  and ν_*n*_^*s*^ with  and .

## 3 Numerical Examples and Discussion

Results of several benchmark calculations on few-electron systems (details of which are given in the Appendix) demonstrate the viability of the aforedescribed five-step reconstruction of the spherically symmetric on-top two-electron density from the NOs and their occupation numbers. In the case of the ground-state electronic wave function of the helium atom, the NOs with  and sufficiently large  accurately reproduce the value of Φ_2_(0), the relative error  at *r* = 0 amounting to 2.74%, 0.47%, and 0.18% for , , and , respectively. For higher angular momenta, the corresponding values of  read 3.48%, 1.00%, 0.55% ; 3.40%, 1.14%, 0.75% ; and 3.36%, 0.62%, 1.32% .

Similar calculations for the ground state of the lithium atom yield estimates of Φ_2_(0) that deviate from its actual value by a few percent, i.e., by 4.59%, 1.81%, and 4.55% for , , and  in the case of  and *s* = α. This level of accuracy is maintained throughout other combinations of the angular momentum and spin, for which the respective values of  equal 4.59%, 0.12%, 1.95% ( and *s* = β); 5.29%, 2.99%, 2.39% ( and *s* = α); 4.70%, 1.80%, 2.74%  and *s* = β); 4.66%, 2.77%, 1.92% ( and *s* = α); 3.98%, 2.23%, 1.73% ( and *s* = β); 4.55%, 2.77%, 1.89%  and *s* = α); and 3.97%, 2.51%, 1.87% ( and *s* = β).

**Figure 2:**
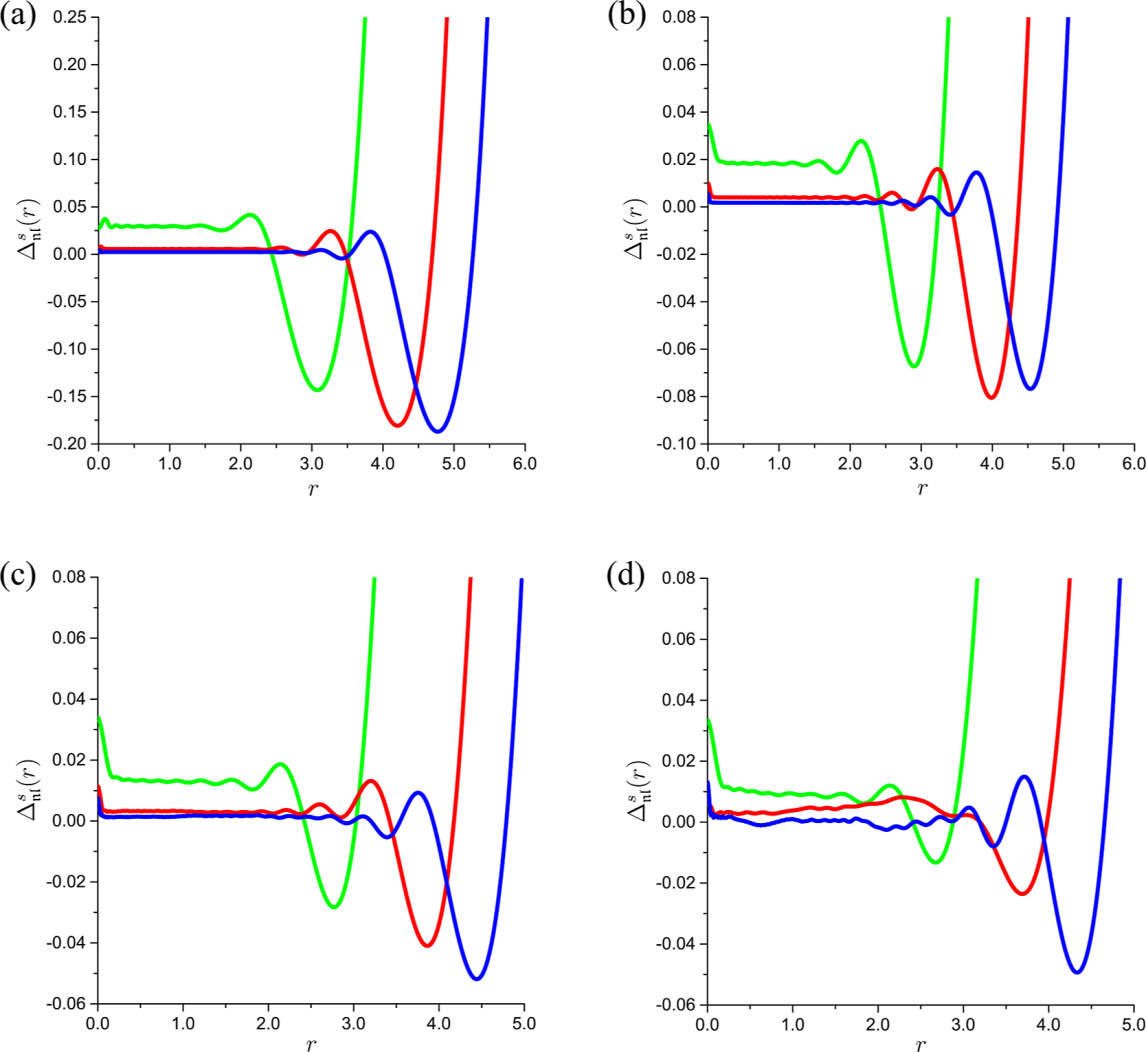
The relative deviations  of the interpolants  from the on-top two-electron density Φ_2_(*r*) of the ground state of the helium atom vs *r* for  (green),  (red), and  (blue): (a) , (b) , (c) , and (d) .

**Figure 3:**
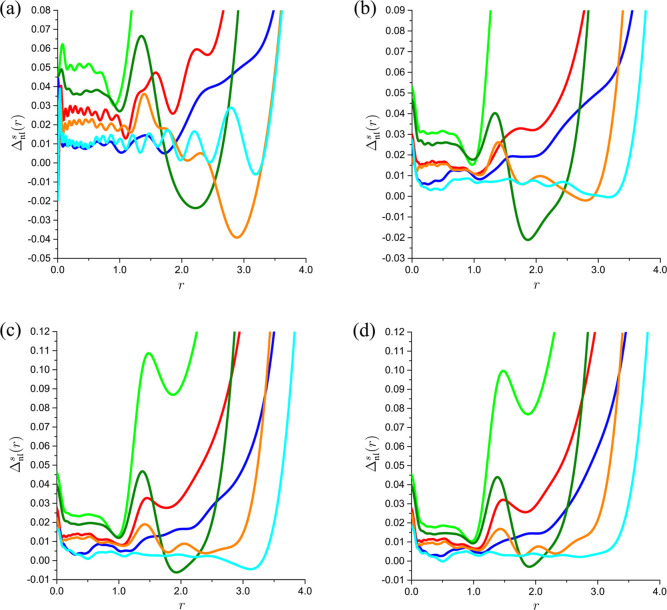
The relative deviations  of the interpolants  from the on-top two-electron density Φ_2_(*r*) of the ground state of the lithium atom vs *r* for  (green),  (olive),  (red),  (orange),  (blue), and  (cyan): (a) , (b) , (c) , and (d) .

Inspection of Figures 2 and 3 reveals a general pattern in the dependence of  on *r*. At small distances from the nucleus, the relative deviations of the interpolants  from Φ_2_(*r*) exhibit plateaus with superimposed oscillations. The radial extents of these plateaus, beyond which the values of  grow rapidly with *r*, increase with . At first glance, this rapid growth may raise concerns about the accuracy of the reconstructed Φ_2_(*r*) being seriously degraded. However, this accuracy degradation is observed for the values of *r* at which the on-top two-electron densities are already very small and thus of no practical significance. For example, Φ_2_(*r*) pertaining to the ground state of the helium atom decays rapidly from ca. 1.868 73 at *r* = 0 to ca. 1.050 23 × 10^–3^ at *r* = 1 and then further to ca. 7.716 30 × 10^–7^ at *r* = 2, whereas the onsets of the accuracy degradation occur at *r* > 2 (Figure 2). Similar conclusions can be drawn for the ground state of the lithium atom, for which Φ_2_(0) ≈ 34.071 9, Φ_2_(1) ≈ 3.817 05 × 10^–4^, and Φ_2_(2) ≈ 2.672 32 × 10^–7^.

**Figure 4:**
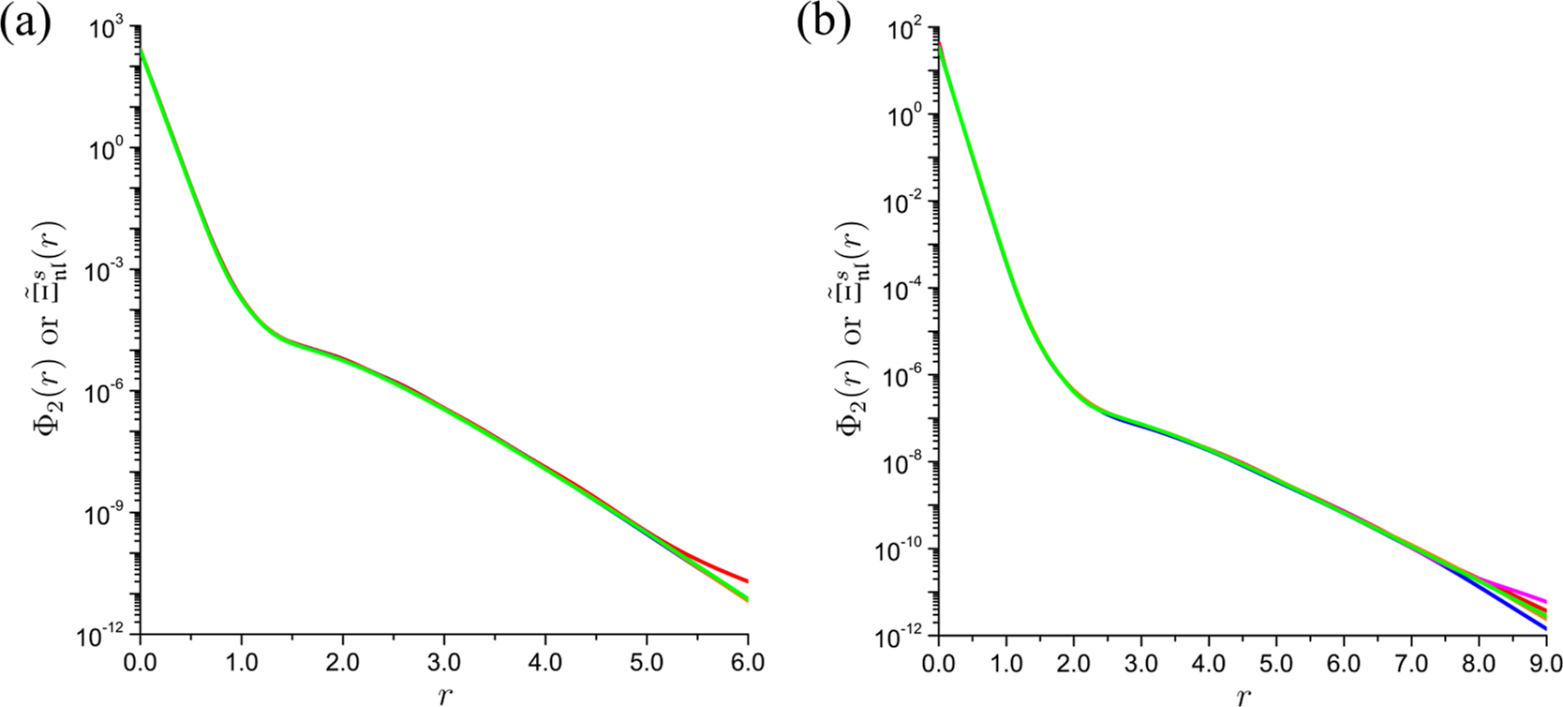
Comparisons of the ground-state on-top two-electron densities Φ_2_(*r*) (green) of (a) the beryllium atom and (b) the lithium anion with the respective interpolants  derived from the 30th NOs with  (red),  (blue),  (magenta), and  (orange).

Despite providing accurate estimates of Φ_2_(*r*) only over limited (though sufficient from a practical standpoint) ranges of *r*, the interpolants  track the overall dependence of Φ_2_(*r*) on *r* with impressive fidelity. For example, in the case of the ground state of the beryllium atom, the interpolants derived from the 30th NOs with , , and  reproduce the evolution of Φ_2_(*r*) with *r* (including the distinct slopes within the core and valence regimes, the regions of convexity and concavity, and the inflection points that separate them) in such a detail that their logarithmic-scale plots over the range 0 ≤ *r* ≤ 6 are not visually discernible from that of Φ_2_(*r*), whereas appreciable deviations in the plot for the corresponding interpolant with  appear only around *r* ≈ 5, which implies a satisfactory reproduction of the values of Φ_2_(*r*) spanning almost 12 orders of magnitude (Figure 4a). For the isoelectronic lithium anion, these deviations emerge at *r* ≈ 7, i.e., in the region where Φ_2_(*r*) is ca. 3 × 10^11^ times smaller than at *r* = 0 (Figure 4b). The same features are present in the analogous plots (which are not included here for the sake of brevity) pertaining to the helium and lithium atoms in their ground states.

## 4 Conclusions

The above examples reveal both the strengths and limitations of the present formalism. On one hand, it allows the approximate reconstruction of Φ_2_(*r*) solely from the gradients and Laplacians of the NOs at their nodal surfaces. This feature makes it universally applicable to all correlated levels of theory that produce sufficiently accurate one-electron properties via genuine 1-matrices (note that the respective two-electron densities often do not even correctly normalize to the number of electron pairs^4^). On the other hand, its accuracy is determined by a trade-off between two opposite trends affecting the error in the computed Φ_2_(*r*). The first of these trends stems from the asymptotic nature of the reconstruction itself, whose accuracy increases with the ordinal number *n* of the underlying *exact* NO, whereas the second one originates from the worsening of the accuracy of the *approximate* counterpart of that orbital upon *n* becoming comparable with the size *M* of the finite one-electron basis set employed in its computation. Although the overall error is bound to decrease with *M*, establishing general rules guiding the optimum values of *n* that maximize the accuracy of Φ_2_(*r*) for various basis sets will require investigations of mostly empirical nature that fall outside of the scope of this paper.

In conclusion, spatial derivatives of the NOs at their nodal surfaces contain approximate information about the on-top two-electron density Φ_2_(*r*). This information, whose accuracy increases with the number of nodal surfaces, allows the reconstruction of Φ_2_(*r*) up to a multiplicative constant that can be retrieved from an identity involving the orbital in question and its occupation number. This reconstruction provides a new consistency check for electronic structure formalisms, such as the one-electron reduced density matrix theory and related approaches,^25^ that employ the NOs as primary quantities.

## Appendix

### Details of the Calculations

In the case of the ground state of the helium atom, the NOs and their occupation numbers have been derived from a highly accurate 2354-term wave function with calculations involving bases comprising 500 one-electron functions.^26^ The calculations on the ground state of the lithium atom have involved a highly accurate wave function constructed from 5896 explicitly correlated Gaussians, from which the NOs and their occupation numbers have been computed with basis sets comprising 160 (320 for ) Gaussian primitives. In the analogous calculations on the ground states of the beryllium atom and the lithium anion, 5896 explicitly correlated Gaussians have been employed in conjunction with 100 (200 for ) Gaussian primitives. A Mathematica notebook illustrating the reconstruction of the on-top two-electron density is available at https://repod.icm.edu.pl in a file indexed with the title of the present paper.
